# Regulation of Cholesterol Metabolism by Phytochemicals Derived from Algae and Edible Mushrooms in Non-Alcoholic Fatty Liver Disease

**DOI:** 10.3390/ijms232213667

**Published:** 2022-11-08

**Authors:** Yahav Eilam, Noam Pintel, Hamdan Khattib, Natalie Shagug, Raged Taha, Dorit Avni

**Affiliations:** 1 Sphingolipids, Active Metabolites, and Immune Modulation Laboratory, MIGAL—Galilee Research Institute, Kiryat Shemona 1101600, Israel; 2 Departmnet of Biotechnology, Faculty of Science and Technology, Tel Hai College, Kiryat Shemona 1220800, Israel

**Keywords:** phytochemicals, active compounds, algae, mushrooms, fungi, cholesterol, inflammation, NAFLD, NASH, sustainable

## Abstract

Cholesterol synthesis occurs in almost all cells, but mainly in hepatocytes in the liver. Cholesterol is garnering increasing attention for its central role in various metabolic diseases. In addition, cholesterol is one of the most essential elements for cells as both a structural source and a player participating in various metabolic pathways. Accurate regulation of cholesterol is necessary for the proper metabolism of fats in the body. Disturbances in cholesterol homeostasis have been linked to various metabolic diseases, such as hyperlipidemia and non-alcoholic fatty liver disease (NAFLD). For many years, the use of synthetic chemical drugs has been effective against many health conditions. Furthermore, from ancient to modern times, various plant-based drugs have been considered local medicines, playing important roles in human health. Phytochemicals are bioactive natural compounds that are derived from medicinal plants, fruit, vegetables, roots, leaves, and flowers and are used to treat a variety of diseases. They include flavonoids, carotenoids, polyphenols, polysaccharides, vitamins, and more. Many of these compounds have been proven to have antioxidant, anti-inflammatory, antiobesity and antihypercholesteremic activity. The multifaceted role of phytochemicals may provide health benefits to humans with regard to the treatment and control of cholesterol metabolism and the diseases associated with this disorder, such as NAFLD. In recent years, global environmental climate change, the COVID-19 pandemic, the current war in Europe, and other conflicts have threatened food security and human nutrition worldwide. This further emphasizes the urgent need for sustainable sources of functional phytochemicals to be included in the food industry and dietary habits. This review summarizes the latest findings on selected phytochemicals from sustainable sources—algae and edible mushrooms—that affect the synthesis and metabolism of cholesterol and improve or prevent NAFLD.

## 1. Introduction

Due to its role in different metabolic diseases, interest in cholesterol is growing. Cholesterol is essential for cells, both as a structural element and as a participant in various metabolic pathways. Accurate regulation of cholesterol is important for normal lipid metabolism in the body. Disruptions of cholesterol homeostasis have been linked to various metabolic diseases, such as type 2 diabetes, atherosclerosis, heart disease, hypercholesterolemia, and even liver diseases, such as non-alcoholic fatty liver disease (NAFLD) [1]. NAFLD is a broad term for a variety of liver diseases caused by Western diets based on high fat and high sugar. The main characteristic of NAFLD is a high accumulation of fat in the liver, which can lead to non-alcoholic steatohepatitis (NASH), a severe form of fatty liver disease that involves inflammation of the liver and may progress to liver failure due to cirrhosis and fibrosis [2]. The overall prevalence of NAFLD worldwide has been recently estimated at 32.4%, increasing significantly over time from 25.5% in 2005 to 37.8% in 2016 [3]. The therapeutic status of NAFLD today includes several treatments, such as antibiotics, Farnesoid X receptor (FXR) agonists, and probiotics, none of which provide a full response. Furthermore, many clinical trials in recent years have failed to provide the desired results. Therefore, NAFLD remains a disease with no satisfactory therapeutic response [4].

Animal cells synthesize cholesterol from acetyl coenzyme A (acetyl-CoA) via more than 20 enzymatic subreactions [5]. In addition, cells receive cholesterol from the circulating blood in the form of mainly low-density lipoprotein (LDL) containing apolipoprotein B. LDL particles enter the cells via the LDL receptor (LDLR) located on the cell surface and are hydrolyzed to free cholesterol in the cell lysosome [6]. Cholesterol concentration in the cells is tightly regulated by various mechanisms related to transcriptional and post-transformation levels [7]. Several genes and transcription factors have been linked to the synthesis and regulation of cholesterol, one of the most prominent being the sterol regulatory element-binding protein (SREBP) family. SREBP transcription factors are encoded by SREBF1 and SREBF2. SREBP-2 activates genes involved in cholesterol synthesis, such as those encoding 3-hydroxy-3-methylglutaryl-coenzyme A reductase (HMGCR), LDLR and mevalonate kinase (MVK), among several others (Table 1). In addition, several microRNAs (miRNAs) are also involved in the synthesis and regulation of cholesterol, including miR-122, miR-33, and miR-370 [8]. These miRNAs regulate the expression of a number of genes responsible for the control of cholesterol levels, such as HMGCR, LDLR, MVK, sterol cleavage-activating protein (SCAP), the phosphoribulokinase (PRKA) family, ATP-binding cassette subfamily A member 1 (ABCA1) and subfamily G member 1 (ABCG1), and cytochrome P450 family 7 subfamily A member 1 (CYP7A1) [9]. Changes in these miRNAs have been reported to contribute to the development or worsening of NAFLD—one of the most common diseases in the western world—caused by the activation of HMGCR. Understanding the relationship between cholesterol metabolism and NAFLD may have preventive or therapeutic benefits [9].

Many studies have demonstrated the diverse effects of phytochemicals on metabolic diseases, cholesterol intake, and dyslipidemia [10]. Phytochemicals are a rich source of bioactive compounds and have anti-inflammatory, antioxidant, and immunomodulatory properties. In addition, different phytochemicals have been shown to have a positive effect on LDL levels and to help prevent the oxidation of lipids in the blood [11].

Worldwide changes in recent years are affecting global supply chains, food security, and nutritional intake. Factors such as climate change, the COVID-19 pandemic, and the current war in Europe are raising the need for phytochemicals from sustainable sources. Algae and edible mushrooms are well-known for their rapid growth while requiring fewer natural resources than higher plants [12,13]. Mushrooms play beneficial roles in improving soil health and increasing agricultural efficiency; they also play a beneficial role in biodiversity and the health of ecosystems and humans alike and have enormous potential for creating food with minimal waste of resources [14]. Algae are also considered a prominent and highly sustainable source of food ingredients. Microalgae have photoautotrophic capacity and yield more vegetation than other, more conventional crops; in addition, they do not need land for cultivation, and they contribute significantly to CO_2_ absorption [15]. Thus, including sustainable sources such as algae and mushrooms in man’s diet has great potential to sustain the global food system.

This review will focus on the effects of alga- and edible mushroom-based phytochemicals on cholesterol metabolism and its relationship to NAFLD development.

## 2. Cholesterol Metabolism, Biosynthesis, and Related Genes

About 20–25% of the cholesterol produced by the body daily is produced in the liver; other cholesterol-producing tissues are the adrenal glands, intestine, and reproductive organs [16]. Cholesterol is an important lipid molecule that serves many biological functions. It is essential for the formation of steroid hormones and bile salts and for the transfer of lipids in the blood. In addition, it is involved in maintaining the membrane’s permeability and fluidity and in the modulation of transmembrane signaling pathways [17].

In the blood, there are several types of cholesterol-carrying lipoproteins. These are, from least to most dense: chylomicrons, very low-density lipoproteins (VLDLs), LDLs, intermediate-density lipoproteins (IDLs), and high-density lipoproteins (HDLs) [18]; the more fat and less protein contained by the lipoprotein, the lower its density. Cholesterol appears in these lipoprotein particles as either free cholesterol or cholesterol-ester, to which a fatty acid is attached [19]. For cholesterol transfer to a cell, the lipoprotein particles attach to receptors on the target cell surface through apo-lipoproteins, used as ligands. Chylomicrons, the cholesterol-carrying particles with the lowest density, carry fats that have been absorbed in the intestine to the muscle tissue, as well as to other tissues that require fatty acids for energy production or fat storage [20]. Cholesterol that is not consumed by the muscle or other tissues remains in the remnants of the cholesterol-rich chylomicrons; these are absorbed by the liver. The liver produces VLDL particles which contain excess triglycerides and cholesterol that are not needed by the liver for the synthesis of bile acids [21]. During their passage through the blood, IDL particles (average density lipoproteins) are formed, which contain a higher percentage of cholesterol [22]. Some of the IDL particles can be absorbed by LDL receptors on the surface of liver cells, whereas the others continue to lose triglyceride molecules in the blood until they become LDL particles with the highest cholesterol concentration [23]. In addition to LDL, HDL is the good cholesterol that transports cholesterol from peripheral tissues to the liver; the liver removes it from the body. Higher HDL levels are linked to a lower chance of heart disease and stroke [24]. The HDL receptor facilitates reverse cholesterol transport. It enables the delivery of lipids to and from HDL lipoproteins by mediating the transfer of cholesterol from the plasma membrane to the HDL lipoprotein. Therefore, LDL particles are the main carriers of cholesterol in the blood, with each particle containing approximately 1500 cholesterol-ester molecules [25]. The LDL envelope contains only one molecule of apolipoprotein B100, which is recognized by LDLRs in the peripheral tissues. The LDL–LDLR complex is introduced into the cell through endocytosis to form an endosome particle, which later fuses with a lysosome; the cholesterol-ester molecules are hydrolyzed in the lysosome by the enzyme lysosomal acid lipase [26]. The free cholesterol is released inside the cell and can be used for biosynthesis of the cell membrane; if it is not needed, it can undergo re-esterification for storage inside the cell until it is needed [27].

**Figure 1:**
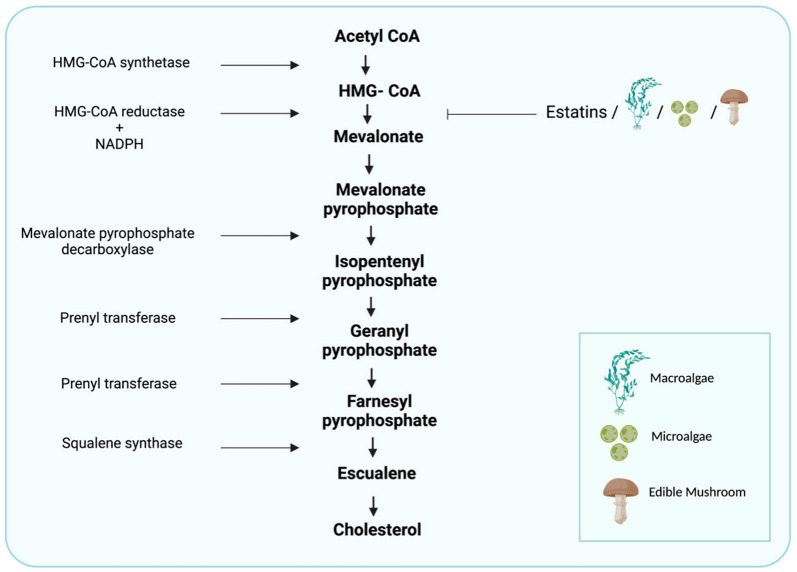
Cholesterol-synthesis pathway. The diagram includes the most relevant enzymes, the mediators of synthesis, and the point at which statins and algal- and edible mushroom-based phytochemicals interfere with the metabolism of HMG-CoA. (Created with BioRender.com).

Cholesterol biosynthesis involves enzymes located in the cytoplasm, microsomes (endoplasmic reticulum [ER]), and peroxisomes. The process starts with an acetyl-CoA molecule and usually occurs in the liver cell’s ER [28]. The first step in cholesterol synthesis produces the intermediate mevalonate, whose synthesis is a committed and rate-limiting step in cholesterol formation. In this reaction, two molecules of acetyl-CoA condense to form acetoacetyl-CoA; catalyzed by 3-hydroxy-3-methylglutaryl-coenzyme A (HMG-CoA) synthase, this molecule condenses with a third acetyl-CoA molecule to form HMG-CoA. The latter is then reduced to mevalonate by NADPH, catalyzed by HMGCR [17]. This is the main regulatory point in cholesterol synthesis that reduces HMG-CoA to mevalonate. Mevalonate then goes through a series of reactions, ultimately yielding cholesterol [17] (Figure 1). The synthesis of HMGCR is controlled by SREBPs, a family of integral ER proteins that regulate the transcription of various genes involved in the cellular uptake of cholesterol and its metabolism. After HMGCR’s synthesis, the SREBPs form complexes with SCAP. The insulin-induced genes (INSIGs), which are related to SCAP, are also involved in the SREBP process. Dissociation of INSIGs from this complex allows its transfer from the ER to the Golgi apparatus [17]. The SREBP–SCAP–INSIG complex is the central regulator of cholesterol levels through the following process. Once the cell’s cholesterol level is high, the binding of cholesterol to SCAP stabilizes the SREBP–SCAP–INSIG complex, and this blocks movement to the Golgi, which causes a decrease in nuclear SREBP and suppresses transcription of the relevant genes, which causes a delay in cholesterol synthesis [29]. Once the level of cholesterol in the cell is depleted, INSIG1 dissociates from the SCAP–SREBP complex, and the latter is transported to the Golgi apparatus [30]. An excessive increase in cholesterol level is associated with a higher risk of developing heart disease, stroke, and peripheral vascular disease. High cholesterol is also linked to diabetes, high blood pressure, liver diseases, and metabolic diseases associated with oxidative and ER stress [31]. In addition to the transcriptional regulation of genes involved in cholesterol metabolism, miRNAs serve as gene regulators, mainly after their transcription [32]. The miRNAs are small non-coding RNA molecules of about 22 nucleotides in length that affect the post-transcriptional expression of genes by modulating the stability of their mRNA or its translation into a protein [33]. The involvement of miRNAs in cholesterol metabolism and their role in regulating gene expression have only just begun to be investigated [9]. Studies by Kathryn et al. showed the involvement of several miRNAs—including miR-122, miR-33, and miR-370—in cholesterol metabolism through the regulation of expression of several genes involved in the control of cholesterol levels such as HMGCR, LDLR, MVK, SCAP, the PRKAs family, ABCA1, ABCG1 and CYP7A1 [9], as well as a potential role in the development of liver diseases. Several miRNAs have been shown to participate in the development of NAFLD, such as miR-34a, miR-122, miR-21, and miR-33 [34].

Cholesterol synthesis occurs in most cells but mainly in hepatocytes and enterocytes. There are several mechanisms related to cholesterol absorption in these latter cells. More than 30 chemical reactions are needed for the biosynthesis of cholesterol, which occurs in the ER. The first two chemical reactions are reversible reactions and are catalyzed by the enzymes thiolase and HMG-CoA synthase, leading to the condensation of two acetate molecules to form acetoacetyl-CoA [35]; this then condenses with a third acetate molecule to form HMG-CoA. Another reaction then occurs, catalyzed by the enzyme HMGCR—a transmembrane protein of the ER that reduces HMG-CoA to mevalonate [35,36]. HMGCR is a key player in cholesterol synthesis. The transcription of this enzyme is responsible for limiting the rate of cholesterol synthesis and is in equilibrium with SREBP-2, which means that its expression is controlled by the concentration of cholesterol in the cells [37].

**Figure 2:**
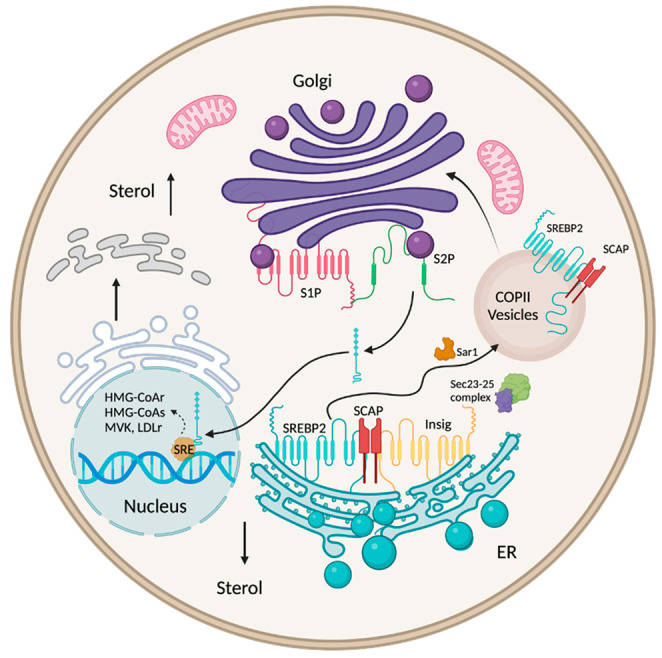
Regulation of cholesterol biosynthesis. SREBP-2 is the main regulator of cholesterol biosynthesis and is responsible for increases in blood cholesterol; it is attached to the ER membrane through its interaction with SCAP and INSIG1. When cholesterol levels decrease, INSIG1 is degraded, and the remaining SREBP-2–SCAP complex is inserted into the COPII vesicle in a process driven by GTPase and with the help of a protein complex consisting of Sar1 and Sec23–25. The SREBP-2–SCAP complex is transported to the Golgi by the COPII vesicle, where it is degraded by proteases S1P and S2P. This causes the release of the active domain of SREBP-2, which is transferred to the nucleus and binds to the SRE, resulting in the transcription of genes involved in cholesterol synthesis: *HMGCR*, *HMG-CoA synthase*, and *MVK*, as well as LDLR, which is responsible for cholesterol uptake. (Created with BioRender.com (accessed on 1 September 2022)).

SREBP-2 is a regulatory protein involved in the third feedback mechanism of cholesterol biosynthesis regulation. This protein is the central regulator of genes involved in the synthesis of cholesterol [38,39]. Located in the ER membrane, SREBP-2 is a double-helix transmembrane protein with two domains: an amino-terminal domain and a carboxy-terminal domain facing the cytosol [40]. The amino terminus includes an active domain that is transported to the nucleus and binds to the sterol regulatory element (SRE) responsible for the activation and translation of genes related to cholesterol synthesis. SREBP is located on the ER membrane connected to two other receptors: SCAP and INSIG, which together form a complex. This complex can only be transported upon dissociation of INSIG1, which is degraded [40] by ubiquitin; the resultant SREBP-2–SCAP complex is inserted into a vesicle called coat protein II (COPII), coated by the proteins Sec23, Sec24, and Sar1 complex [40,41]. COPII is then moved to the Golgi apparatus and is degraded by site-1 protease (S1P) and site-2 protease (S2P). This releases the amino-terminal active domain of SREBP-2 and SCAP [42]. SREBP-2 then moves to the nucleus and binds to the SRE, inducing the transcription of genes involved in cholesterol synthesis, such as HMGCR, HMG-CoA synthase, and MVK, as well as LDLR, which is responsible for cholesterol uptake [38] (Figure 2).

## 3. Cholesterol Metabolism and Pathophysiology of NAFLD

NAFLD is the most common liver disease worldwide, with a prevalence of 20–30% in the general population of western countries. In NAFLD, excess fat accumulates in the liver (to more than 5% of the liver’s weight) of individuals who drink little or no alcohol [43]. The most common form of NAFLD is non-alcoholic fatty liver (NAFL), where the fat accumulates in the liver cells; although this condition is not normal, it probably does not damage the liver in and of itself, and there is no inflammation [44]. However, 10–20% of NAFL patients will progress to a more severe state of the disease termed NASH, in which fat accumulation activates inflammatory processes. NASH can lead to severe scarring of the liver and cirrhosis. Cirrhosis occurs when the liver is significantly damaged and can lead to liver cancer [45]. Recent studies have shown that a disruption in the balance of cholesterol metabolism in the liver causes the accumulation of lipids and, consequently, liver toxicity and NAFLD. Free cholesterol may accumulate in NAFLD due to its upregulation in the input pathways or a decrease in its elimination. Studies have shown that NAFL/NASH patients have increased expression of HMGCR [46,47].

**Figure 3:**
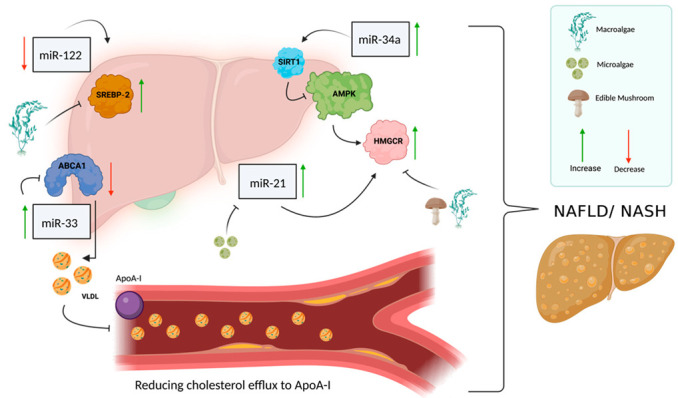
miRNAs involved in cholesterol metabolism and NAFLD. Several miRNAs are involved in cholesterol metabolism and NAFLD, such as miR-122, miR-34a, miR-33 and miR-21. miR-122 is responsible for the regulation of SREBP-2 in its inactive form and is normally expressed at a high level in the liver, but in NAFLD patients, there seems to be a decrease in miR-122 expression level, which causes an increase in the transcription factor SREBP-2. miR-34a has a key role in the development of NAFLD through its involvement in the dysregulation of cholesterol metabolism. miR-34a is responsible for the suppression of sirtuin 1 (encoded by SIRT1), which in turn is responsible for regulating AMP kinase (AMPK) activity, which regulates HMGCR phosphorylation. When miR-34a levels increase, SIRT1 suppresses AMPK, which causes a decrease in HMGCR levels. As a result of this series of actions, the regulation of cholesterol metabolism is impaired, and there is an increase in cholesterol accumulation in the liver, which contributes to the development of NAFLD. miR-33 is overexpressed in the liver of NAFLD patients and functions as a regulator of lipid and insulin metabolism; when it is overexpressed, it suppresses ABCA1 expression. Suppression of ABCA1 results in a reduction in cholesterol efflux to ApoA-I, which is a key player in the regulation of reverse cholesterol transport. High levels of miR-21 also characterize NAFLD/NASH patients; a high level of miR-21 causes the accumulation of fats in the liver and leads to an increase in HMGCR expression and impaired regulation of cholesterol metabolism. (Created with BioRender.com (accessed on 1 September 2022)).

Increasing evidence indicates that a change in miRNAs results in the constant activation of HMGCoAr. Studies have shown that several miRNAs are associated with the development of liver diseases and NAFLD [48], including miR-34a, miR-122, miR-21, and miR-33, which have a regulatory role in hepatic functions [34]. miR-122 is the first miRNA to be described in humans for its effect on the liver; it is highly expressed in the liver, representing about 70% of the total miRNA pool in that organ. miR-122 modulates the inactive form of SREBP-2, and a significant decrease in miR-122 has been found in patients with NASH, potentially explaining the increase in SREBP-2 [49]. In NAFL/NASH patients, there is an increase in SREBP-2 expression; its overexpression is not found in obese patients or patients with other liver diseases, such as hepatitis C. SREBP-2 may be activated in NAFL/NASH by an additional molecular pathway that is responsible for the increase in hepatic free cholesterol levels, but it is independent of the pathway related to changes in cellular cholesterol [50]. miR-34a has an important role in NAFLD development: it is involved in the dysregulation of cholesterol metabolism because it causes the suppression of sirtuin 1 (encoded by *SIRT1*), a molecule that is responsible for the regulation of AMP kinase (AMPK) activity, which regulates HMGCR phosphorylation [51,52]. Increasing evidence suggests miR-34a silencing is a novel therapeutic approach for the treatment of NAFLD. miR-33 has increased expression in the liver of NAFLD patients [53]. It regulates the metabolism of lipids and insulin-signaling pathways. Its overexpression in NAFLD patients greatly suppresses the expression of ABCA1, promotes cellular cholesterol efflux, and mediates the efflux of cholesterol to lipid-poor apolipoproteins (apoA-I). ABCA1 repression decreases cholesterol efflux to ApoA-I, which has a major role in regulating reverse cholesterol transport [32]. miR-21 expression level is also increased in NAFL/NASH patients. miR-21 induces hepatic lipid accumulation through interaction with several factors, such as SREBP-1, HMGCR, and fatty acid binding protein 7 (FABP7) [54] (Figure 3).

The liver plays a central role in cholesterol homeostasis. In NAFL/NASH patients, in addition to the increase in cholesterol synthesis due to the activation of HMGCR, there are changes in the pathways involved in cholesterol elimination. A decrease in the expressions of CYP7A1 (responsible for cholesterol catabolism and synthesis of bile acids) and ABCG5/G8 (responsible for cholesterol secretion into bile) has been reported [51]. Therefore, it is clear to us that in NAFL/NASH patients, there is a basic increase in the synthesis and accumulation of free cholesterol in the liver and a decrease in cholesterol elimination.

## 4. Western Diet and Relation to Cholesterol Metabolism and NAFLD

Obesity is an increasing health concern worldwide that is influenced by the western diet and lack of physical activity. High cholesterol content in the western diet and lifestyle plays a central role in the development of many metabolic diseases, such as type 2 diabetes, cardiovascular diseases, and NAFLD.

The western diet was originally attributed to the United States, but it has migrated to many countries in the world, including Asian countries [42,55]. This diet was developed with the aim of getting the population to regularly consume certain food products by adding taste-stimulating and addictive ingredients such as sugar, salt, and processed fats, mostly soy and canola, in very large quantities [56]. The western diet also includes high consumption of red meat, processed meat, packaged food, fried food, high-fat dairy products, eggs, processed grains, potatoes, corn and high-fructose corn syrup, high-sugar drinks, and food additives (as classified by E numbers, such as titanium dioxide) [57]. This diet also minimizes and/or eliminates the consumption of plant-based foods, meaning less dietary fiber and fewer vitamins, minerals, and antioxidants [58].

In recent years, the relationship between nutrition and metabolic diseases has been well established. Today, it is known that consumption of a high-fat diet (HFD) that raises blood cholesterol (LDL) levels contributes to the development of fatty liver diseases such as NAFLD [59]. Moreover, a recent study has shown that a toxic combination of cholesterol and dietary fat affects components of the immune system in the liver, such as macrophages. By using an in vivo model, that study presented the cascade of events occurring in the immune system in the liver, which ultimately contributed to the development of local inflammation and scarring of the liver, similar to that which occurs in non-alcoholic steatohepatitis (NASH). Mice were fed an HFD, and this dietary composition had synergistic harmful effects on the genes that regulate inflammation and scarring of the liver [60].

## 5. Effect of Cholesterol-Synthesis Inhibitors on NAFLD

Today, statins are useful drugs for the inhibition of cholesterol synthesis. They include lovastatin, simvastatin, pravastatin, fluvastatin, atorvastatin, and cerivastatin. These six drugs inhibit the enzyme HMGCR, resulting in decreased synthesis of cholesterol in human hepatocytes [61]; on the other hand, they cause increased transcription of LDLR and the absorption of LDL cholesterol (LDL-c) in the liver [62]. Statins are indeed effective for most people, but they are also associated with muscle pain, mental fogginess in some individuals, sleep problems, digestive problems, a decrease in blood platelet count, and in rare cases, they can cause liver damage [63]. Studies have shown that statin therapy seems safe for use in patients with NAFLD. However, it is not yet known whether they should be used for the specific treatment of NAFLD.

**Table 1:** Transcription factor, enzyme, receptors, and their associated genes involved in cholesterol metabolism and NAFLD.

Transcription Factor/Enzyme/Receptor	Related Gene	Cholesterol Metabolism	NAFL/NASH	References
SREBPs (SREBP-2)	SREBF1/SREBF2	Transcription factors activate genes that are involved in the synthesis of cholesterol, such as HMGCR, LDLR and MVK	Increases gene expression levels. NAFLD progression and deterioration	[64] [47]
HMG-CoA	HMGCR	Converts HMG-CoA to mevalonate, which is an enzyme that limits cholesterol biosynthesis	Increases gene expression levels. NAFLD progression and deterioration	[65] [51]
LDLR	LDLR	Receptor; binds to LDL particles, which serve as the main carrier of cholesterol in the blood	Decrease gene expression levels. NAFLD progression and deterioration	[66] [51]
MVK	MVK	Catalyzes the phosphorylation of mevalonate to mevalonate 5 phosphate, a key step in cholesterol biosynthesis	Increases gene expression levels. NAFLD progression and deterioration	[67] [65] [51]
SCAP	SCAP	Part of the SREBP–SCAP complex that causes translocation of SREBP from the ER to the Golgi apparatus, resulting in the regulation of cholesterol levels	No data. NAFLD progression and deterioration	[68]
AMPK	α—PRKAA1, PRKAA2 β—PRKAB1, PRKAB2 γ—PRKAG1, PRKAG2, PRKAG3	A kinase-type enzyme that plays a role in cells’ energy homeostasis, causes the oxidation of fatty acids in the liver, inhibits cholesterol and triglyceride, consists of several subunits	Decreases gene expression levels. NAFLD progression and deterioration	[68,69]
ABCA1	ABCA1	Transporter, cholesterol-efflux mediator for apolipoprotein (apoA-I), maintains cholesterol homeostasis in the body	Decreases gene expression levels. NAFLD progression and deterioration	[70] [51,70]
ABCG1	ABCG1	Transporter, excess cholesterol flows from the cells to HDL particles, resulting in reverse cholesterol transport, and considered the only factor that removes cholesterol from the body	Decreases gene expression levels. NAFLD progression and deterioration	[71] [51]
CYP7A1	CYP7A1	A receptor that accelerates the initial step in cholesterol catabolism and bile acid synthesis	Increases gene expression levels. NAFLD progression and deterioration	[72] [51]

## 6. Phytochemical Effects on Cholesterol Metabolism and NAFLD

**Figure 4:**
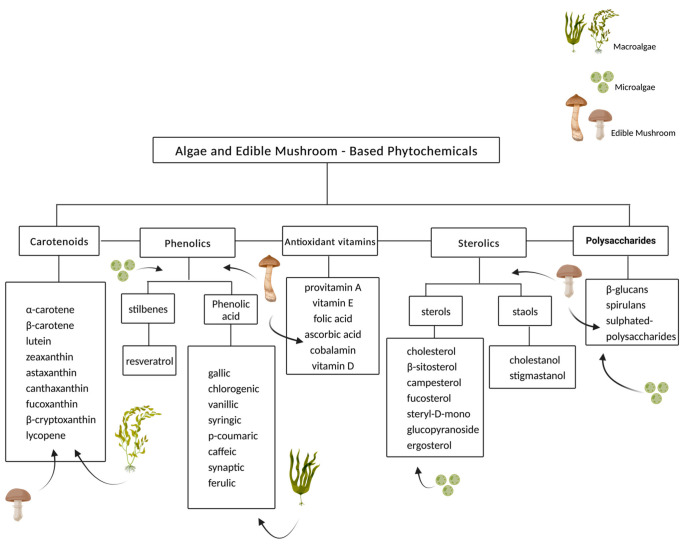
Scheme of alga- and edible mushroom-based phytochemicals. Phytochemicals are secondary metabolites and bioactive compounds naturally found in plants and organisms such as microalgae, macroalgae and edible mushrooms. Among phytochemicals, there are carotenoids, sterols, polysaccharides, and phenols that have biological activity that affects human health. Many phytochemicals act as antioxidants; others succeed in suppressing harmful processes in the body’s cells, preventing the proliferation of cancer cells, regulating lipid levels and having anti-inflammatory activity. (Created with BioRender.com (accessed on 1 September 2022)).

Phytochemicals are secondary metabolites and bioactive compounds primarily derived from fruit, vegetables, cereals, and other natural sources. Beyond their use in basic foods as nutrients, they may offer health benefits by reducing cholesterol metabolism and the risk of chronic diseases such as NAFLD due to their anti-inflammatory and immunomodulatory [73] properties. Several phytochemicals exert their beneficial effects by lowering circulating cholesterol levels or preventing lipid oxidation. Laka et al. showed that phytochemicals inhibit the expression of enzymes and transcription factors associated with cholesterol metabolism, resulting in lower liver cholesterol levels. Moreover, phytochemicals decrease the level of LDL, suggesting the prevention of cholesterol accumulation. The major classes of phytochemicals include vitamins, polysaccharides, polyphenols, terpenoids, steroids, alkaloids, tannins, saponins, and thiols [11] (Figure 4).

### 6.1. Clinical Significance of Phytochemicals in Therapeutic Approaches to NAFLD

NAFLD is characterized by the accumulation of excess lipids in the liver tissue, leading to inflammatory and oxidative damage [74]. There are a number of clinical trials exploring phytochemicals as a therapeutic approach for NAFLD. One such study evaluated a curcumin-based polyphenol of nutritional origin for its effect on lipid properties and its anti-inflammatory and antioxidant activity [75]. The effectiveness and safety of supplementation with encapsulated curcumin-based polyphenol (phytosomal form) were assessed in 102 NAFLD patients in different stages of the disease (1–3, classified according to liver ultrasound examination). The patients received 1000 mg per day of curcumin in phytosomal form for 8 weeks. The curcumin-based polyphenol was found to be associated with a decrease in body mass index, an improvement in liver ultrasound findings showing a decrease in hepatic fat levels, and a reduction in serum aspartate aminotransferase and alanine aminotransferase levels. Curcumin-based polyphenol appeared to improve liver fat levels, and its use appeared to be safe and well-tolerated during the trial [76]. In another randomized controlled clinical trial in which children with NAFLD (classified by biopsy) participated, the addition of 2000 IU per day of vitamin D for 6 months was associated with significant improvement in liver steatosis and lobular inflammation, as determined by liver biopsy taken after treatment [77]. Another active clinical trial illustrating the potential of phytochemicals for the treatment of NAFLD bears mentioning: a randomized, double-blind, placebo-controlled, parallel-group clinical trial examining the potential of vitamin E and docosahexaenoic acid ethyl ester (DHA-EE) administration for NAFLD patients is still in progress [78,79].

### 6.2. Vitamins

#### 6.2.1. Vitamin E

The most significant lipid-soluble antioxidant is vitamin E. It effectively prevents many disease processes linked to oxidative stress [80]. Due to its solubility, bile salts and pancreatic secretion are required for intestinal absorption [81]. When vitamin E enters the intestine, it forms micelles that are passively diffused and partially mediated by cholesterol membrane transporters through the membrane [82]. Some of the vitamin E moves to HDL, and the rest remains in the chylomicron [83] remnants. In the liver, these remnants bind to α-tocopherol, lipoproteins, and triglycerides. They form VLDL, which is then transported to the plasma. VLDL is separated into VLDL, HDL, and LDL [84], and therefore, vitamin E may act as an inhibitor of cholesterol biosynthesis. Sato et al. investigated the clinical effect of vitamin E on NAFLD patients and showed significant improvement in their liver function. The vitamin E supplement decreased liver fat by 0.54 U/L [85]. As the number of patients with hepatic steatosis rises, vitamin E supplementation may be an adequate treatment opportunity for preventing the deterioration of NAFLD [86].

#### 6.2.2. Vitamin B12

Vitamin B12 is an essential cofactor in important methylation processes related to DNA and cell metabolism [87]. Studies have also demonstrated that a low level of vitamin B12 is related to a high total cholesterol level together with an increased ratio of LDL to HDL in the serum, causing the biosynthesis of cholesterol and homocysteine in adipocytes. Moreover, a low level of vitamin B12 increases the expression of SREBPs and genes responsible for the biosynthesis of cholesterol and leads to a reduction in the levels of AdoMet to AdoHcy and hypomethylation of the regulatory regions of *SREBF1* and *LDLR* [88].

### 6.3. Polyphenols

Polyphenols have been described as plant secondary metabolites. They are usually found in foods such as cereals, fruit, vegetables, wine, coffee, tea, and many other derived foods. Their most salient health benefits are associated with their antioxidant and anti-inflammatory properties. The advantages of polyphenols as a prebiotic substrate are attributed to the intestinal microbiota’s ability to metabolize phenolic compounds [89], such as pomegranate peel polyphenols, their main component punicalagin, and its metabolite pomegranate ellagic acid. These polyphenols were tested in human hepatocyte cell line L-02; all of them reduced lipids and enhanced cholesterol metabolism, with ellagic acid being the most effective, followed by peel polyphenols. The molecular mechanism may be via activation of the target gene peroxisome proliferator-activated receptor gamma (*PPARγ*) and then upregulation of the expression of the downstream genes *ABCA1* and *CYP7A1* via a PPARγ ABCA1/CYP7A1 cell-signaling pathway, leading to cholesterol metabolism in liver cells. In addition, pomegranate peel polyphenols promote cholesterol metabolism in liver cells, so they could be used in a drug regimen or as a functional food-based material and may be a more appropriate prevention and treatment option for diseases related to excessive cholesterol accumulation [90].

### 6.4. Flavonoids

Flavonoids are a group of natural substances with variable phenolic structures, including flavonols, flavanols, flavanones, flavones, and isoflavones. They are found in fruit, vegetables, grain, bark, roots, stems, flowers, tea and wine and are well known for their beneficial effects on health [91].

#### 6.4.1. Flavonols

Flavonols are widely distributed in plants and in large amounts in fruit and vegetables. Aside from their antioxidant effects, they interfere with many biochemical signaling pathways [92]. Quercetin is a flavonol found in capers. Chambers et al. showed that in rats, quercetin increases Cyp7a1 in the liver and liver X receptor α (LXRα) mRNA and protein, along with the increased secretion of bile acid (BA) and hepatic BAs. Quercetin also increased the expression of ABCG1 mRNA and protein, suggesting its involvement in the regulation of hepatic cholesterol efflux. Another flavonoid, kaempferol, increased hepatic CYP7A1, fecal cholesterol, and BAs, possibly via its binding to LXRα [93].

#### 6.4.2. Flavanols

Flavanols (also termed flavan-3-ols) are a family of bioactive compounds found in cocoa, red wine, green tea, red grapes, berries, and apples. These potent antioxidants scavenge free radicals. They may exhibit other activities as well, including modulation of intracellular signaling, effects on membrane fluidity, and regulation of cytokine release or action [94]. The catechin flavonoids have been found to increase CYP7A1 mRNA levels, with epicatechin gallate showing the most marked induction. Conversely, epigallocatechin gallate is a known activator of Farnesoid X receptor signaling, which suppresses CYP7A1 expression. Human hepatocytes treated with epigallocatechin gallate in the absence of serum showed a reduction in CYP7A1 gene expression [93].

#### 6.4.3. Flavanones, Flavones, and Isoflavones

The flavone naringin occurs naturally in citrus fruit [95]. It has been shown to induce LDLR and CYP7A1 expression in liver cells through nuclear factor kappa-light-chain-enhancer of activated B cells (NF-κB) and extracellular signal-regulated kinase (ERK) signaling pathways, as well as PPARy. The isoflavone puerarin (from arrowroot) has been shown to increase hepatic CYP7A1 expression and suppress hepatic cholesterol. In mice and rats, isoflavone-rich extracts increased hepatic Cyp7a1 expression and BA secretion, respectively. Soy milk and fermented soy milk, both of which contain isoflavones, bioactive proteins and peptides, reduced liver cholesterol and triglyceride levels and increased hepatic *Cyp7a1* expression in rats [93].

### 6.5. Anthocyanins

Anthocyanins suppress lipid accumulation in adipocytes as a result of a broad inhibition of the transcription factors that regulate lipogenesis [96]. One of the most common anthocyanidins (aglycons of anthocyanins) is cyanidin, a compound that is naturally abundant in fruit and vegetables [97]. It affects all three PPAR subtypes, showing the greatest affinity for PPARα [98]. Cyanidin-3-O-β-glucoside (C3G) activates LXR-ATP-binding cassette transporter-dependent cholesterol efflux and phosphorylation of cellular AMPK in human hepatoma (HepG2) cells [99]. C3G was shown to decrease the expression of adipogenic transcription factors involved in hepatic lipid metabolism, including SREBP-1c, CCAAT enhancer-binding protein α (C/EBPα), and PPARγ, in both HepG2 cells and the liver of supplemented mice [100]. The effect of anthocyanins on suppressing SREBP-1c and C/EBPα gene expression has also been verified in other studies [101,102,103].

### 6.6. Iridoids

Iridoids are a type of monoterpenoid found in a wide variety of plants, typically as glycosides. Gentiopicroside, a subgroup of seco-iridoid compounds, ameliorated dyslipidemia and improved nerve blood flow by regulating the PPARγ/AMPK/acetyl-CoA carboxylase (ACC) signaling pathway in a rat model. Moreover, in both acute and chronic alcohol-induced mouse hepatosteatosis (the latter stage of alcoholic liver disease), gentiopicroside decreased SREBP-1 upregulation and PPARα downregulation via the activation of AMPK. A decrease in SREBP-1 expression was observed in a nonalcoholic fatty liver disease model [96].

### 6.7. Palmitoylethanolamide

Palmitoylethanolamide regulates LXR activity, which causes activation of PPARα by PPARγ, in addition to the downstream *ABCA1*. In contrast, it does not affect retinoid X receptor α. Palmitoylethanolamide promoted cholesterol removal by increasing fecal bile acid and upregulating the two pathways LXR/PPAR–ABCA1 [104].

### 6.8. Alkaloids

Alkaloids are important secondary metabolites with therapeutic properties. They can prevent the onset of various degenerative diseases via free-radical scavenging or binding with the oxidative reaction catalyst [105].

#### 6.8.1. Nuciferine

Nuciferine is an active aporphine alkaloid that has a significant effect on reducing intracellular triglyceride levels and improving lipid metabolism in 3T3-L1 adipocytes [106]. Nuciferine prevented hepatic steatosis in golden hamsters fed with HFD [107]. Supplementation with this compound significantly alleviated serum and hepatic lipid levels and enhanced expression levels of PPARα and carnitine palmitoyl transferase 1 (CPT1), which contributed to increased fatty acid oxidation. In addition, nuciferine treatment significantly reversed the increase in hepatic levels of proteins SREBP-1c and fatty acid synthase (encoded by *FASN*) caused by the HFD, indicating that nuciferine can suppress the development of hepatic steatosis induced by an HFD [106,108].

#### 6.8.2. Betaine

Betaine notably enhances hepatic steatosis in C57BL/6J mice through AMPK activation and SREBP-1c downregulation [109]. It also suppressed triglyceride accumulation in the liver by reducing methylation of PPARα, thus increasing its expression [110]. Similar results were found in rats fed an HFD, where betaine enhanced hepatic lipid export and fatty acid oxidation by increasing the expression of PPARα and CPT1 [111]. In addition to this, betaine has shown a promotion in the absorption of fatty acids through increasing the expression of genes involved in fatty acid transport, such as cluster of differentiation (FAT/CD36)/fatty acid translocase, fatty acid transport protein (FATP1) and FABP3 in the muscle tissue of finishing pigs [106].

### 6.9. Omega-3 Fatty Acids

The polyunsaturated omega-3 fatty acids regulate transcription factors associated with lipid metabolism in the liver, resulting in higher oxidation of fatty acids and the regulation of downstream proinflammatory genes [112]. Omega-3 fatty acids have also been shown to reduce circulating triglycerides by reducing the hepatic secretion of VLDL cholesterol or increasing chylomicron metabolism. Chan et al. showed that fish oil supplementation lowers triglycerides (−18%), VLDL apolipoprotein B (−20%) and hepatic excretion of VLDL apolipoprotein B (−29%) compared to supplementation with placebo [113]. This effect resulted in a 35% reduction in the synthesis of triglycerides and an increase in the mitochondrial oxidation of fatty acids [114].

## 7. Effects of Plant-Based Active Compounds on Cholesterol Metabolism and NAFLD

### 7.1. Microalgae

Microalgae are a diverse class of eukaryotic and prokaryotic unicellular organisms that are predominantly autotrophic. Only a few species have been thoroughly explored: *Dunaliella*, *Chlorella*, *Isochrysis*, *Nannochloropsis*, *Chlamydomonas*, *Haematococcus*, and *Spirulina*, although the number of microalgal species has been estimated at around 200,000 (according to AlgaeBase, www.algaebase.org (accessed on 1 September 2022)). Due to their well-balanced chemical components, microalgae are one of the most viable sources of nutrients and bioactive substances for new food products that can be utilized to improve the nutritional content of foods. In fact, adding microalgal biomass to food products is an intriguing way to add biologically active supplements to the diet, such as vitamins A, B1, B2, B6, B12, C, and E, proteins with essential amino acids, polysaccharides, monounsaturated and polyunsaturated fatty acids (MUFAs and PUFAs, respectively), nucleic acids, minerals such as iodine, potassium, iron, magnesium, and calcium [115,116], and several bioactive compounds such as dietary fiber, polyphenols, carotenoids, phycobiliproteins, and polysaccharides [117] (Figure 4). The complement of bioactive compounds differs among species growing in different environments—temperature, light conditions, pH, CO_2_ level, nutrients, and salts [118]. Due to their antioxidant, antimicrobial, anti-inflammatory, anticarcinogenic, antidiabetic, antihypertensive, antihyperlipidemic, and antiobesity activities, these molecules have potential health applications [119,120]. Microalgae are applied in the cosmetic, food, and pharmaceutical industries and are considered to be therapeutic agents for a number of medical conditions due to their ability to produce a variety of primary and secondary metabolites [121]. One of the many health benefits of microalgae is balancing cholesterol metabolism by regulating several genes that also affect liver function, which plays a key role in cholesterol homeostasis [122].

#### 7.1.1. Microalgal Phytochemicals Affecting Cholesterol Metabolism in the Liver and NAFLD

The major phytochemicals—important secondary metabolites—are alkaloids, flavonoids, terpenoids, carotenoids, glycosides, phenols, and sterols [123]. Phytochemicals are essential to several processes, serving as inhibitors of oxidation, cancer, viruses, bacteria, and enzymes. There are about 4000 phytochemicals of both terrestrial and marine origin that have established health benefits, with marine phytochemicals having higher potential biological activity than terrestrial ones [124]. With their high concentration of phenols and terpenes, microalgae are a significant source of phytochemicals and nutritional supplements [125].

Carotenoids are lipid-soluble pigments that are primarily responsible for living organisms’ distinctive purple, red, orange, and yellow colors [120]. They are divided into carotenes (lycopene and β-carotene) and xanthophylls (lutein, zeaxanthin and astaxanthin) [126].

##### Microalgal Astaxanthin

Yoshida et al. investigated the administration of astaxanthin on serum HDL cholesterol (HDL-c) and found several mechanisms, including reverse cholesterol transport from peripheral tissues to the liver, where astaxanthin can be used to treat impaired lipid metabolism and improve the serum lipid profile in humans, including an increase in HDL-c and significant negative risk for several diseases [127]. In patients with NAFLD and NASH, miR-21 was more highly expressed than the other miRNAs [54], and its increase was in direct response to fast food and, therefore, to increases in the hepatic level of unsaturated fatty acids [128]. Shatoor et al. investigated the effect of astaxanthin on NAFLD in relation to miR-21. In rats fed an HFD, they examined whether astaxanthin can improve hepatic steatosis by modifying the nuclear factor erythroid-2-related factor 2(Nrf2)/miR-21 axis [128]. Further studies proved that Nrf2—a fundamental antioxidant transcription factor [129] and a dominant inhibitor of miR-21 [130]—was elevated and activated, mediating the hypolipidemic and antisteatotic actions of astaxanthin [131].

Astaxanthin was also investigated as a preventive of NAFLD through its decrease in SREBP1/2 expression, which inhibits oxidative stress and inflammation, and through activation of PPARα, which significantly promotes fatty acid oxidation and suppresses the production of triglycerides. SREBP1/2 are the primary transcriptional factors promoting the production of triglycerides and cholesterol [128]. Shatoor observed a significant reduction in serum levels of triglycerides (38%), cholesterol (33%), and LDL (32%), and hepatic levels of triglycerides (13%) in rats fed a standard diet with astaxanthin treatment compared to those fed a standard diet alone (*p* < 0.05). In contrast, the HFD + astaxanthin group significantly outperformed the HFD group in terms of liver weight (107% improvement), serum triglycerides (91.3%), cholesterol (106%), and LDL-c (60%) levels, as well as hepatic triglycerides (88%), cholesterol (75%), and free fatty acid (84%) levels (*p* < 0.0001). The percentages of improvement were calculated as follows: (levels for HFD + astaxanthin − levels for HFD]/[levels for HFD − levels for standard diet). Shatoor et al.’s results suggest that astaxanthin reduces hepatic lipid production via an Nrf2-dependent increase in hepatic free fatty acid oxidation and suppression of cholesterol and triglyceride synthesis. When Shatoor et al. investigated the changes in the expression of miR-21 and lipogenic genes, they observed a high positive association between the mRNA levels of hepatic miR-21 and SERBP-1/2 (r^2^ = 0.886 and r^2^ = 0.8682, respectively). These findings show that astaxanthin reduces Srebp-1 and Srebp-2 expression while increasing PPAR expression in the livers of HFD-fed rats. These results imply that astaxanthin may inhibit miR-21 in an Nrf2-dependent manner to prevent the initiation and progression of NAFLD in rats fed an HFD diet [128] (Figure 3).

##### Microalgal Fucoxanthin

Antioxidant, anticancer, anti-inflammatory, and antiobesity benefits are only a few of the health-promoting properties of fucoxanthin [132]. Fucoxanthin significantly influences the enzymes that control cholesterol levels and effectively lowers triglyceride levels in the liver and adipose tissue [133]. Fucoxanthin also impacts the expression of genes involved in lipid metabolism, including those for fatty acid synthase, stearoyl-CoA desaturase-1 (SCD1), C/EBP, PPAR, and SREBP [134]. Koo et al. evaluated body and organ weight following supplementation with *Phaeodatylum* extract (PE), known as a potential source of fucoxanthin, and found uncoupling protein 1 (UCP1) upregulation in brown adipose tissue and PPAR downregulation, indicating the mechanism by which PE regulates lipid metabolism [135]. Those authors also determined that PE controls lipid metabolism via PPAR and UCP1 and, as a result, has an antiobesity effect. Koo et al. also explored the effect of PE on serum lipid parameters to evaluate its effect on lipid metabolism and liver damage. PE treatment resulted in a dose-dependent reduction in LDL levels and could also reduce lipid metabolism dysfunction and liver damage. Livers of the HFD-fed group had more lipid droplets than those of the normal diet group, but PE treatment reduced lipid droplet number and size. Triglyceride level was also reduced in the PE-treated group. These results show that PE affects hepatic steatosis mainly by reducing excessive fat accumulation [134,135]. Mayer et al. examined whether lipids and carotenoids from the microalga *Phaeodactylum tricornutum* can prevent the cell characteristics of NAFLD. The human hepatocarcinoma cell line HepG2, frequently used to research lipid metabolism and metabolic abnormalities in the liver, including those related to NAFLD [136], served as the cell model. Cells were treated with 250 mM palmitate for 24 h—a procedure typically applied to promote lipotoxicity and hepatic steatosis in HepG2 cells—to induce NAFLD [137,138]. They showed that carotenoid extract (CE) inhibits the increase in total cholesterol, cholesterol-ester, and triglycerides and reduces the lipid droplet accumulation induced by palmitate. The major bioactive component of CE, fucoxanthin, may control the expression of lipid metabolism-related enzymes such as ACCα, fatty acid synthase, diacylglycerol O-acyltransferase 1, and SCD1; additionally, it could decrease the mRNA levels of acyl-CoA:cholesterol acyltransferase 1/sterol O-acyltransferase 1 (ACAT1/SOAT1) in HepG2 cells [139,140]. Finally, CE elevated CPT1A’s mRNA level, suggesting that fucoxanthin functions by promoting the beta-oxidation pathway to reduce lipid droplet accumulation and production. Furthermore, Mayer et al. explain that CE appears to have modifying effects on lipid metabolism by increasing the mRNA levels of CPT1A, a gene associated with fatty acid oxidation, and decreasing the mRNA levels of lipogenesis genes, indicating that fucoxanthin CE may serve as a powerful anti-NAFLD therapy.

##### Microalgal Polysaccharides

Microalgal polysaccharides may constitute another category of natural lipid-regulating compounds, particularly considering the non-digestible polysaccharides from a variety of food sources that have been recognized for their lipid-regulating properties [141]. Xylose, glucose, and galactose are the major sugars in the heteropolymeric cell wall polysaccharides [142]. The combination of glucuronic acid and sulfate groups gives these molecules a negative charge [143]. Dvir et al. used *Porphyridium* sp. Polysaccharides to investigate their therapeutic effect on the lipid metabolism of hypercholesterolemic rats fed diets rich in cholesterol [142]. The results were similar to those in other studies investigating the effect of microalgae on lipid metabolism [144,145,146,147] and showed a decrease in the accumulation of hepatic cholesterol, plasma triglycerides, and VLDL cholesterol. In another study, animals fed soluble fibers to reduce cholesterol were shown to have higher levels of HMGCR, which is essential for endogenous cholesterol formation [148]. Compared to control animals, hypercholesterolemic rats fed microalgal polysaccharides at levels of 5% or 10% had increased levels of HMGCR.

Wan et al. analyzed hepatic mRNA and protein levels of genes associated with the AMPK pathway, an important factor in regulating glycolipid metabolism, to examine the mechanism governing microalgal polysaccharides’ effects on the hypolipidemic activity in diet-induced rats [149]. After treatment with microalgal polysaccharides, hepatic steatosis and lipid droplets decreased, along with liver triglycerides, total cholesterol and body weight [149]. Therefore, microalgal polysaccharides could serve as a good source for improving lipid metabolism by regulating triglyceride and LDL-c metabolism. It was also observed that in comparison to the HFD group, microalgal polysaccharide significantly increased AMPK mRNA expression, which in turn reduced HMG-CoA and SREBP-1 levels and inhibited the synthesis of fatty acids [149] (Figure 3).

Gou et al. examined the potential effects on the lipid metabolism of polysaccharides isolated from *Chlorella pyrenoidosa* (CPC) and *Spirulina platensis* (SPC) in mice fed an HFD with a reference compound of β-glucan [150]. A significant role of the ligand-dependent transcription factor PPAR was identified in the late phase of adipocyte development [151]. Through the activation of PPAR expression, the production of an endogenous PPAR ligand, and the regulation of the expression of several crucial genes for lipid biosynthesis, the transcription factor SREBP-1c plays an essential role in the development of adipocytes [152]. Gou et al. used Western blot analysis to show that the hepatic PPAR and Srebp-1c levels in HFD control mice were significantly higher than those in low-fat diet control animals, indicating that HFD-fed mice had higher levels of adipogenesis. Oral administration of β-glucan, CPS, and SPS significantly inhibited the HFD-induced rise in both adipogenic transcription factors, supporting their contribution to lowering lipogenesis in mice fed the HFD [150] and therefore characterizing them as a potential source for improving lipid metabolism. Fucoidan, a low-molecular-weight polysaccharide found in algae, reduced hepatic accumulation of triglycerides and cholesterol, as well as total cholesterol, in mice. In addition, this polysaccharide elevated the level of the anti-inflammatory adiponectin, an adipose hormone associated with inflammation, while down-regulating various proinflammatory cytokines and transcription factors. Fucoidan treatment also resulted in the activation of hepatic AMPK signaling. According to the findings of Zheng et al., fucoidan polysaccharide prevents NAFLD in mice by inducing the AMPK signaling system [153].

##### Microalgal Phytosterols

Phytosterols are secondary metabolites of cholesterol-like substances found naturally in plants. They reduce plasma LDL-c and cholesterol absorption [154]. As secondary metabolites of steryl glycosides, steroid glycosides are a class of major natural compounds with nutritional and pharmaceutical value [155]. Steryl glycosides are a group of sterol metabolites in which a sugar compound is bound to the hydroxyl group of the sterol. In plants, steryl-D-mono glucopyranoside is the most common steryl glycoside [156]. Steryl glycosides have significant biological activities, such as controlling lipid metabolism, developmental processes, and host defenses against pathogens [157]. A great source of steryl glycosides is microalgae. Recently, it was revealed that the marine microalga *Pavlova viridis* has a 5.234 mg/kg dry weight of steryl glycosides. Microalgae can produce a more diverse range of sterols than higher plants [158]. However, there is considerable variation in steryl glycoside composition across various microalgae [159]. In general, phytosterols are considered to lower cholesterol levels by competitively inhibiting cholesterol absorption and modifying the genes in enterocytes and hepatocytes that are involved in cholesterol metabolism [160]. According to a recent study, dietary phytosterols increase the expression of the hepatic *HMGCR* in response to the low hepatic cholesterol levels brought on by the phytosterol-induced reduction in intestinal cholesterol levels [161]. Feng et al. examined, by quantitative PCR, the expression of genes involved in lipid and bile acid metabolism to understand the mechanisms governing phytosterol’s reduction of hepatic lipid buildup and bile acid pool size. Their results showed that HMGCR mRNA expression in the liver was significantly reduced in a high-fat western diet, but phytosterols stopped the reduction. PPAR expression also showed a significant increase upon phytosterol supplementation. Feng et al. also reported that long-term phytosterol consumption inhibits NAFLD development via cholesterol reduction in the liver [160]. In addition, phytosterols have been shown to be novel treatment compounds for hepatic fibrosis, LDL concentration reduction, improving insulin resistance, down-regulating systemic inflammation, and increasing circulation of endothelial progenitor cells in patients with NAFLD [160,162].

##### Microalgal Phenolics

The largest class of phytochemicals are polyphenols, many of which were discovered in foods derived from plants [163]. These plant-derived secondary metabolites classified as phenolic compounds share an aromatic ring with one or more hydroxyl groups [164]. Plant sources have been identified with several phenolic substances, such as simple phenols, flavonoids, lignins and lignans, tannins, xanthones, and coumarins [165]. Safafar et al. investigated the total phenolic content in several different microalgal species using the Folin–Ciocalteu method and revealed a significant difference among them, which may depend on their growth conditions and oxidative stress. The highest concentration was found in *Desmodesmus* with 7.72 mg/g [166]. As a defense strategy against oxidative stress brought on by excess reactive oxygen species, polyphenols are essential compounds that support and enhance the activities of antioxidant vitamins and enzymes. Therefore, polyphenol-rich diets have been associated with a number of health advantages against, for example, cancer, inflammation, cardiovascular diseases [163,167] and liver injuries [168].

Several signaling pathways, including those that increase fatty acid oxidation by upregulating PPAR, decrease lipogenesis by downregulating SREBP-1c and activating AMPK and increase antioxidant defense through the Nrf2 pathway. Polyphenols may protect against hepatocyte injury caused by NAFLD. Various polyphenols are considered to act through AMPK regulation, especially with c-Jun N-terminal kinase (JNK) and p38 MAPK, which have been suggested as viable pathways for active therapy for NAFLD [168]. SREBP-1c has also been recognized as a target in NAFLD therapy. Its increase in the liver promotes fatty liver, and studies have shown that phenols may inhibit SREBP-1c by downregulating its protein and gene expression [169].

### 7.2. Macroalgae

Macroalgae, commonly known as seaweed, are large, macroscopic algae that resemble grass. Due to their size, macroalgae have the ability to eliminate harmful toxins due to their large surface area, which allows biological absorption of toxic compounds from the environment. In addition, they have essential ingredients with health benefits, such as proteins, polysaccharides and omega-3 fatty acids [170] (Figure 4). Macroalgae are classified into brown algae, green algae and red algae. Red and brown algae are considered marine algae, whereas green algae may also be found in freshwater, such as lakes and rivers [171]. Macroalgae may contain large amounts of tocopherols, a group of compounds that have vitamin E activity and are powerful antioxidants [172], as well as additional antioxidants such as vitamins A and C. Moreover, macroalgae contain a number of protective pigments and secondary metabolites [173]. These macroalgal compounds have been shown in recent studies to have anti-inflammatory, antioxidant, anticarcinogenic, antidiabetic, anti-Alzheimer, and antimicrobial activity, as well as antihyperlipidemic and antiobesity effects [174]; they play a significant role in health promotion.

#### 7.2.1. Macroalgal Phytochemicals Affecting Cholesterol Metabolism in the Liver and NAFLD

Cholesterol is an essential factor for cell homeostasis. Cholesterol homeostasis is a tightly regulated process, and any imbalance can result in hypercholesterolemia, including high levels of total cholesterol, LDL-c, or triglycerides, and even a decrease in HDL-c [175].

In vivo studies have reported the hypocholesterolemic effect of different macroalgal species [176,177,178,179]. In most studies, algae rich in phytochemical extracts were used as a dietary supplement, resulting in decreased total cholesterol, triglyceride and LDL-c levels and increased HDL-c levels. Those effects on blood lipid levels might be explained by the modification of cholesterol biosynthesis due to a modulatory effect on the high-affinity receptor of lipoprotein metabolism. In addition, macroalgae have the reported ability to absorb cholesterol and excrete it from the body through the feces. A recent in vivo study of rats fed a 20% HFD and an additional 2.5% of extracts from the macroalgae *Ecklonia arborea* or *Silvetia compressa* showed different results for the two algae with respect to cholesterol levels and liver histology [180]. *S. compressa* caused an 18% decrease in total cholesterol, whereas *E. arborea* increased cholesterol levels by 5.8% compared to the control rats. In addition, the group fed the *S. compressa* extract had lower levels of LDL-c. Liver histology showed a reduction in steatosis in the group fed *S. compressa* vs. *E. arborea* extract. This suggests *S. compressa* as a resource for the reduction of serum cholesterol and liver tissue damage [180].

##### Macroalgal Polysaccharides

Polysaccharides derived from plant foods are major components of the human diet and serve as major sources of energy in all diets [181]. NAFLD is caused by the accumulation of fat in the liver and is associated with a disorder in cholesterol metabolism [182]. Previous studies have shown that polysaccharides from the alga *Enteromorpha prolifera* lower cholesterol levels in diabetic rats [182]. A study based on those results examined the possible effect of *E. prolifera* polysaccharides on the development of NAFLD and its underlying mechanisms. These polysaccharides, supplemented at 200 mg per kg body weight, significantly reduced liver weight and also decreased mRNA and protein expression of HMGCR in the liver [182]. They also suppressed the regulatory element of sterol-binding protein 2—a key transcription factor in cholesterol metabolism and regulator of HMGCR expression. In light of these results, *E. prolifera* polysaccharides may provide a functional food that can prevent NAFLD [182]. Moreover, microalgal polysaccharides can reduce blood lipid levels and total cholesterol and simultaneously increase HDL-c levels [183,184]. In addition, they reduce the absorption of cholesterol in the body by binding the diet-supplied cholesterol compounds and excreting them from the body through the feces [185]. Cholesterol absorption by polysaccharides is affected by the ellagic acid found in large quantities in macroalgae. Sodium alginate in seaweed turns into free ellagic acid, and in this process, it is transformed into a gel that prevents absorption in the small intestine and drives the excretion of cholesterol. In addition, in aged hypercholesterolemia-induced rats that received HFD for 12 weeks together with an aquatic extract of *Ulva fasciata* polysaccharides, the extract showed antihypercholesterolemic properties. Thus, it could be used as a natural lipid regulator [186]. Another study examining the effect of polysaccharides from *U. fasciata* showed significant decreases in total cholesterol, triglycerides and total lipids compared to the untreated rats [187].

##### Macroalgal Fucoxanthin

Seaweed’s antioxidant capacity is known to be due in part to carotenoids. Fucoxanthin [177] is the most abundant carotenoid in macroalgae. Its hypolipidemic effect has been described in various studies, where it lowered the levels of cholesterol and triglycerides in the liver and therefore affected both the absorption of cholesterol in the liver and by inhibiting HMGCR and its synthesis [188,189] (Figure 4). Fucoxanthin also causes the absorption of cholesterol and its excretion through the feces by reducing the mRNA levels of ACAT and increasing those of lecithin cholesterol acyltransferase; these two enzymes are responsible for catalyzing the esterification of free cholesterol to cholesteryl ester [188].

##### Macroalgal Phenolics

The main phenolic compounds in macroalgae, and in particular red macroalgae, are phlorotannins, which make up as much as 25% of the algae’s dry weight. Phlorotannin activity and its role in hypolipidemic activity have not yet been completely described, but it is now known that these compounds have an effect on the biosynthesis and absorption of cholesterol [190]. A phenolic-rich extract from *Fucus vesiculosus* and *Ecklonia cava* was able to inhibit cholesterol absorption, although the mode of action has yet to be clarified [191,192]. Moreover, other studies have shown that *E. cava* can inhibit cholesterol esterase, which limits the absorption of dietary cholesterol [193]. Similarly, phenolic compounds isolated from plants or fruit showed the ability to inhibit NPC1-like intracellular cholesterol transporter 1 (NPC1L1), a transporter that takes part in cholesterol absorption, similar to the HMGCR inhibitor (statin) [194,195]. Ezetimibe Na et al. isolated new anti-lipid compounds from the edible brown macroalga *Ecklonia stolonifera*. They showed that an ethanolic extract enriched with phenolic phlorotannin affected lipid levels in the blood of rats with hyperlipidemia induced by HFD. The results showed reductions in triglycerides, total cholesterol, and LDL-c levels, as well as a significant increase in HDL-c levels.

### 7.3. Edible Mushrooms

In addition to their aromatic taste and culinary properties, edible mushrooms are a rich source of bioactive compounds. Mushrooms have recently become attractive as a functional food and for their potential impact on human health. Many cultures over the years have consumed mushrooms as both food and medicine. Today, mushrooms are consumed as food due to their low-fat content and high nutritional value, as they contain proteins, polysaccharides, polyphenols, fibers, minerals and vitamins [196,197,198] (Figure 4). The bioactive compounds found in mushrooms, in particular edible mushrooms, have anti-inflammatory, anticancer, antioxidant and anticholesterolemic properties [199,200].

#### 7.3.1. Fungal-Based Phytochemicals Affecting Cholesterol Metabolism in the Liver and NAFLD

Studies have revealed a hypocholesterolemic effect of *Pleurotus ostreatus* on rats fed an HFD and those with a hereditary cholesterol disorder [201]. The decrease in cholesterol levels was also demonstrated by a decrease in LDL levels, in addition to the elimination of HMG-CoA reductase activity [202] and a reduction in total cholesterol and triglycerides in the plasma [203,204]. Moreover, an interesting study on the Mexican mushroom *Ganoderma lucidum* provided evidence for its hypocholesterolemic effects in C57BL/6 mice. In that study, a significant reduction was observed in the expression of lipogenic genes, such as HMRC, FASN, SREBP-1C and ACACA, and of genes responsible for the reverse transport of cholesterol, such as ABCG5 and ABCG8, as well as increased expression of LDLR in the liver [205].

Previous studies have shown that vitamin D deficiency is linked to NAFLD, and both conditions are considered risk factors for metabolic diseases [206]. In addition, there is evidence linking vitamin D, NAFLD, and increased cholesterol levels [207].

C57BL/6 mice fed an HFD together with *Lentinula edodes* mushroom extract or with vitamin D-enriched mushroom extract for 25 weeks showed significant attenuation of total body fat accumulation rate, decreasing liver fat content, a significant decrease in serum triglycerides and LDL-c, and an increase in the HDL/LDL ratio. These results indicate that the consumption of mushrooms enriched with vitamin D may serve to treat or prevent the development of NAFLD [208].

##### Fungal Polysaccharides

Polysaccharides are the most common carbohydrates in foods, with a wide variety of physiological functions according to their composition and molecular weight [209].

*Pleurotus ostreatus*, also called the oyster mushroom, is considered one of the most consumed mushrooms in the world, along with the white button mushroom (*Agaricus bisporus*). A particularly notable advantage of this mushroom is the fact that it grows quickly compared to other mushrooms, and in addition, it contains many bioactive compounds, including β-glucans—a polysaccharide derived from D-glucose that is linked by glycosidic bonds, which has been shown to decrease serum LDL-c. *P. ostreatus* contains twice as much β-glucan as *A. bisporus* [210]. *P. ostreatus* is also known for its potent ability to produce lovastatin, a pharmaceutical therapy agent which helps lower cholesterol levels by inhibiting HMG-Co-A reductase. Therefore, consumption of a diet rich in *P. ostreatus* results in lower triglyceride and cholesterol levels. In addition, polysaccharides from *L. edodes* showed a decrease in the expression level of vascular cell adhesion molecule 1 (VCAM-1) mRNA of the thoracic aorta endothelial cells in rats, with increased antioxidant enzyme activity and a significant decrease in total cholesterol, triglycerides, and lipoprotein-cholesterol levels together with inhibition of damage caused by oxidative stress in the blood due to an HFD [211]. Li et al. examined the impact of polysaccharides found in the edible fungus *Grifola frondosa* on potential regulatory mechanisms controlling lipid and cholesterol metabolism in rats [212]. The production of 7-α-hydroxycholesterol, which is crucial for the maintenance of cholesterol levels, is catalyzed by CYP7A1, a critical rate-limiting enzyme in the synthesis of bile acids [213]. Livers of rats supplemented with *G. frondosa* had significantly higher levels of hepatic CYP7A1 mRNA, which may have enhanced the production of bile acids and decreased hepatic triglycerides [212].

Previous research has shown that *Ganoderma amboinense* polysaccharide (GAP) can protect the liver. In a recently published study in which mice fed an HFD were given GAP for 8 weeks, GAP effectively prevented NAFLD development and reduced body weight, liver weight, and blood lipid levels. It appears that GAP promotes the transport of fat in the liver by regulating the content of phosphatidylcholine in the blood and regulates several metabolic pathways that protect the mitochondrial function responsible for the rapid catabolism of liver cell fats. According to these results, it appears that GAP may help prevent and treat NAFLD [214].

##### Fungal Phenolics

Phenols are a large family of organic compounds that are found in plants and fungi. Polyphenols are widely used as antioxidants and have a beneficial effect on human health. Their regular consumption is associated with reductions in several metabolic diseases, including cardiovascular disease, obesity, liver disease, and insulin resistance [215]. Recent studies have reported that a diet rich in polyphenols may play an important role in preventing high cholesterol levels. One example of polyphenols are flavonoids, which improve glucose and lipid metabolism in the liver [216]. *Suillus luteus* and *Boletus badius* are edible mushrooms that are rich in polyphenols [217]. Both polyphenols and dietary fiber from mushrooms can reduce cholesterol and bile acids produced by the liver. Bile acids are secreted into the small intestine to facilitate digestion and absorption of dietary fats. They are reabsorbed by enterocytes and transported back to the liver through the enterohepatic circulation. When polyphenols and dietary fibers mix with bile acids in the enterohepatic circulation, the bile acids are excreted together with cholesterol through the feces. This results in reduced cholesterol levels and has a positive effect on the blood lipid profile [218].

##### Fungal Flavonoids

Flavonoids are found naturally in many plants, as well as in edible mushrooms. Flavonoids are known to have antioxidant activity and a positive effect on metabolic diseases such as type 2 diabetes and inflammatory, heart, and liver diseases [219]. Increased consumption of flavonoids may reduce the risk of many chronic health conditions. *Ganoderma lucidum* is a white Basidiomycete root mushroom that has been eaten in the Far East for many years and is believed to improve health and enhance longevity [220]. *G. lucidum* contains high amounts of flavonoids, such as quercetin and kaempferol [221]. According to Jung et al. [222], a fraction isolated from *G. lucidum* improved non-alcoholic steatosis and the associated complex disorders by inducing energy-metabolizing enzymes. In an in vivo experiment, *G. lucidum* helped reduce the weight of the epididymal and perirenal adipose tissue and reduced serum cholesterol and LDL levels in mice fed an HFD. In addition, *G. lucidum* reduced AMPK and ACC phosphorylation in HFD-fed mouse liver and caused increased phosphorylation of AMPK and ACC in HepG2 and 3T3-L1 cells. Therefore, *G. lucidum* appears to participate in energy metabolism regulation and lipid accumulation directly in adipose tissue and in the liver. Therefore, *G. lucidum* may serve as a treatment or preventive measure for NAFLD or other metabolic disorders [222].

##### Fungal Sterols

Ergosterol is a sterol found in fungal cell membranes; it performs many of the same functions as cholesterol in animal cells or phytosterols in plants. Because many fungi cannot survive without ergosterol, its synthesis-related enzymes have emerged as crucial drug-discovery targets. Ergosterol is a provitamin form of vitamin D2, which is used in human nutrition [223]. Das et al. investigated fat-soluble components extracted from *Agaricus bisporus* (button mushroom) and their biological activities on fat-related metabolic processes and gene expression. The results revealed that treatment with ergosterol at 34.96 mg/g of extract in an HFD group could reduce hepatic cholesterol accumulation, as well as liver triglyceride levels by 26%. The ergosterol extract significantly reduced HMG-CoA and SREBP-2 expression, and the expression of LDLR, which is responsible for hepatic cholesterol clearance, was greatly enhanced. Therefore, these findings may contribute to the use of ergosterols as therapeutic agents for NAFLD and other liver diseases [224].

## 8. Conclusions and Future Directions

Many studies support the claim that metabolic diseases can be prevented by adopting healthy lifestyle habits and reducing risk factors that can lead to an increase in LDL-c levels. Consumption of phytochemicals and biological compounds from plant sources results in better control of blood lipid levels.

Our review examines the positive effects of sustainable sources such as algal- and fungal-based phytochemicals on managing hyperlipidemia and preventing the development of NAFLD, as well as their related effects on the various pathways and genes involved in the development of these conditions.

Phytochemicals regulate lipid metabolism and have multi-target involvement in absorption, transport, elimination, and cholesterol biosynthesis. HMGCR, MVK, LDLR and CYP7A1 remain the key molecules involved in these processes.

In addition, following these processes, transcription factors such as SREBPs take an active part in helping phytochemicals lower lipid levels. The effects of phytochemicals seem promising and emphasize the need for and importance of their use as potential treatments. The algal and mushroom-based phytochemicals described in this review are diverse, including polysaccharides, phenols, sterols, and flavonoids, and are considered safe and well-tolerated in most cases. Phytochemicals can reduce LDL-c levels and prevent NAFLD by regulating various metabolic pathways. In addition, in this review, we described the involvement of a number of miRNAs—miR-122, miR-33, and miR-370—in cholesterol metabolism and the development of NAFLD. Various phytochemicals also seem to have an effect on these components, which can lead to a reduction in blood lipid levels. Dietary fibers, such as the β-glucans found in edible mushrooms and algae, are known to inhibit cholesterol absorption in the intestines, prevent the absorption of bile acids, and affect cholesterol synthesis in the liver to decrease LDL-c levels.

Today, there is a therapeutic gap for patients suffering from hypercholesteremia: 70% of patients do not respond well to statins. In addition, there is no suitable therapeutic approach for NAFLD, which is currently considered the leading chronic liver disease in the western world. The relationship between phytochemicals and these diseases may offer a new direction, with a therapeutic advantage for those suffering from these disorders or as a potential supportive treatment through dietary inclusion.

Plant foods are viewed in a positive light due to their health benefits. It is worth noting that in light of the global challenges facing the world food system, edible mushrooms and algae—which are considered sustainable sources—might be important sources of functional phytochemicals promoting health and wellbeing and enabling food security worldwide. In contrast, there is an issue with microalgae in that only a few species have been approved for human consumption (GRAS): *Arthrospira maxima, Arthrospira platensis, Chlamydomonas reinhardtii, Chlorella protothecoides, Dunaliella bardawil, Haematococcus pluvialis and Prototheca moriformis*. Further studies supporting the importance of additional microalgae-based phytochemicals for cholesterol metabolism and NAFLD could convince the regulators to approve new algae for human consumption.

Despite this limitation, the consumption of algal- and edible mushroom-based phytochemicals is an effective strategy for managing cholesterol metabolism and NAFLD, and these could potentially be used as functional ingredients for the food industry.
